# Pauper Lunacy

**Published:** 1859-07-01

**Authors:** 


### Art. II.—PAUPER LUNACY.

According to returns contained in the "Further Report" of the Commissioners in Lunacy, published in 1847, and in their Eleventh Report, the number of Lunatics and Idiots chargeable to parishes, throughout England and Wales, amounted on the 1st of January 1847 to Eighteen Thousand and Sixty-Five, and on the 1st of January 1857 to Twenty-Seven Thousand Six Hundred and Ninety-Three.

Moreover, according to returns contained in the 12th Report of the Commissioners, it would appear that on the 1st of January, 1857, the number of pauper lunatics (idiots being also included under the term) cared for in public and private asylums amounted to 16,657; and on the 1st of January 1858, to 17,572. At the latter date there was accommodation for 16,231 lunatics in public asylums; and new asylums and additions to existing asylums, since completed or now in progress, will raise the accommodation to 21,048.

Further, from a recently published supplement to the 12th Report we learn that the number of lunatics in workhouses amounted on the 1st of January 1857 to 6800; on the 1st of January 1858 to 7555; and on the 1st of July in the same year to 7666.

These figures merit considerable attention, for they show a very great and even startling increase in the amount of pauper lunacy in the decennial period 1847-57; a persistence of that increase since 1857; and an amount of asylum accommodation, existing and prospective, barely sufficient to meet pressing wants, and totally inadequate for future wants, or, indeed, for the present proper care and treatment (in the most restricted sense) of our pauper lunatics.

The returns for 1847-57 represent solely an increase in amount of *pauper* lunatics, and it does not follow that there has been a proportionate increase of lunacy, or, indeed, any increase at all, among those classes of the population from which pauper lunatics are principally derived. For, since 1847, a vast impetus has been given to the care and treatment of lunatics, by the efforts of the Lunacy Commission, which at the date mentioned had barely got into effective operation; by the erection of public asylums for the reception of lunatics; and by the compulsory activity of parochial authorities in searching for and bringing to light cases of lunacy which ought to be under care. Hence we may infer that one cause of the increase of pauper lunatics has been the greater care devoted to lunatics, in consequence of which many cases hitherto unheeded have been brought to our knowledge; and that, in so far as this cause is operative, the increase does not represent an absolute increase of lunacy among the impoverished classes. The Chairman of the Lunacy Commission, the Right Honourable the Earl of Shaftesbury, in his examination before the recent Parliamentary Commission on Lunatics, seemed to imply that the entire increase of pauper lunatics might be attributed to the cause mentioned. In answer to a question respecting the great apparent increase in the number of lunatics generally, he answered—

"I am satisfied that it was not a positive increase in the actual number of lunatics in proportion to the population. Of course, as the population increases, there must be a certain increase in the number of lunatics, but the lunatics did not, in that time, increase in the ratio of population, but the appearance was owing to the provision having been made for them, and the greater activity of all the authorities to look them up in all directions, and to bring them to the face of day and place them in the receptacles prepared for them; and, I think, I cannot give a better proof of that than this, that when the activity began and all these cases were brought to light, they were found to be not recent cases but they were old-established chronic cases, of very long standing indeed; and it was a very sad thing when Hanwell was extended, and also when Colney Hatch was opened, which we had hoped would be for the reception of recent and curable cases, it was almost instantly filled with old and chronic cases, to the exclusion of the recent and curable cases that might have been brought there, and many of them returned in a state of health to the duties of life."—(Query 49, *Report of the Select Committee on Lunatics*, p. 7.)

In reply to a question if it were his opinion that there had not been an increase of lunacy in the country, his Lordship said, after stating that there was a great difference of opinion upon the point, on account of the imperfection of the data previous to the year 1845 :—

"I will state my opinion, which is pretty well borne out by my brother Commissioners and a good many others, that the increase of lunacy is certainly unquestionable, but it is not by any means in the ratio of the increase of the population. If the population is increasing, it would be certainly supposed that the number of lunatics would be increased; and if it has increased, say, for example, at the rate of 20 per cent., there has not been the same ratio of increase, that is, of 20 per cent."—(Query 51, p. 7.)

Again in his answer to a subsequent query, his Lordship said :—

"With regard to the progress of insanity among the pauper classes, I repeat that I do not believe that it is by any means in proportion to the increase of population."—(Query 59, p. 9.)

Mr. Gaskell, one of the medical Commissioners in Lunacy, in his evidence before the Select Committee, stated, in answer to the question—

"In your opinion does insanity increase; you say that you have brought many cases to light, but does insanity increase more than in proportion to the population?—It increases more than in proportion to the population; but I am inclined to think that that increase is not attributable to an increase in the number of cases of insanity so much as to the increase in their longevity. There is no doubt that, in many of our asylums where the annual mortality was perhaps 12, 14, or 15 per cent. formerly, it is now reduced 10 per cent., but that in a population of 500, 600, or 1000 in a very few years causes an enormous increase to the actual number."—(Query 1435, p. 138.)

If we assume that the increase of pauper lunacy has depended mainly upon the greater care and provision for lunatics, we must also assume that, notwithstanding the period over which that care has prevailed, we have not yet exhausted the number of hitherto unrecognised cases requiring care among the classes of the population from which pauper lunatics are chiefly derived, since the increase of pauper lunatics, between the 1st of January 1857 and the 1st January 1858, in public and private asylums and workhouses amounted to 1670. This being the case it is certainly of considerable importance that we should have some more definite knowledge, than we as yet possess, of the unexhausted substratum of lunacy which these figures indicate. Moreover, if we assume with Mr. Gaskell that a greater duration of life among lunatics in asylums has been operative in swelling the amount of pauper lunacy, we have still no measure of the degree in which this cause has affected the increase. A more prolonged life, and the greater care given to the welfare of lunatics have doubtless contributed in no small degree, and it may be altogether, to occasion the augmentation of pauper lunatics during the ten years 1847-57; but the influence of both these causes can be accurately determined, and we submit that the question is much too important to be left in its present vague and unsatisfactory state.

Again, the Earl of Shaftesbury's opinion that although there is an increase of lunacy among the pauper classes, still that increase is not by any means in proportion to that of the population, cannot be received without considerable hesitation, gratifying though the opinion may be. Until the influence of those causes to which we have referred as affecting the apparent increase of lunacy is determined, we are not in a position to estimate the absolute increase or not from the returns on which such an estimate must at present be based.

The published returns of the Commissioners in Lunacy show that the number of pauper lunatics and idiots, during the ten years 1847-57, increased at the rate of *four per cent per annum*—the increase of *idiots* being at the rate of three per cent; that of *lunatics* at five per cent. The rate of increase of population is 1·220 per cent per annum. These figures show, at the least, an absolute increase of pauperism from lunacy and idiocy (the chief immediate practical question) at a rate that would double the existing amount of pauper lunacy and idiocy in *twenty-five years*. If we need any other evidence of the importance of obtaining some positive knowledge as to the cause or causes of this great rate of increase, we need but turn to the figures showing the present and prospective public asylum accommodation. This is sufficient for 21,048 lunatics, and on the 1st of January 1858, the number of pauper lunatics detained in both public and private asylums amounted to 17,572, being an increase on the previous year of 915 cases. If this rate of increase were to continue, and if the whole of the remaining public asylum accommodation were devoted entirely to pauper lunatics, in about *five* years every asylum would be filled, and we should still lack space for our lunatics. The amount of accommodation in private asylums for pauper lunatics may be regarded as having reached its maximum, for the tendency of legislation is to provide for all pauper lunatics in public asylums, while the number of private asylums is slightly decreasing (*Report of Select Committee*, Qs. 108 and 113); and if it be conceded, as is maintained by many, that the 2467 pauper lunatics contained in private asylums (1st January 1858), and the 7666 lunatics housed in workhouses should be transferred to asylums (and the need which exists for the transference of lunatics in workhouses can scarcely be doubted, as will be presently seen) it will be perceived that with regard to our asylum accommodation we are pretty nigh at a dead-lock.

With these facts staring us in the face the necessity for an accurate knowledge of the amount of insanity in England can hardly be doubted, and the nigh approach of another census year at once points to the best means by which this knowledge can be attained.

In the Census of 1851, inquiry was directed, so far as Insanity was concerned, only to the number of lunatics in asylums and workhouses. The rapidly increasing magnitude of the subject of lunacy will, we trust, induce the Government in 1861 to direct that means shall be taken to ascertain the total number of lunatics, idiots, and imbeciles existing in the kingdom; as well as the circumstances under which they are placed as regards comfort or indigence: and we must earnestly urge the consideration of this important and, we think, indispensable investigation upon all persons who are interested in obtaining effective provision for the care and treatment of lunatics (and who is not?)

The data which would be derived from a systematic authoritative inquiry into the amount of insanity would form the basis for a true knowledge of the progression of mental unsoundness in the kingdom. The same data would, also, serve as a measure by which we might estimate the probable asylum accommodation required, and thus enable us to set a limit to the vast expenditure which is being undergone for the care of lunatics, as well as to judge more clearly of the means most requisite, in addition to existing means, for that purpose.

But we conceive that a specific inquiry is required, not only into the amount of insanity in the kingdom and the circumstances under which it is placed, but also into the causes which foster lunacy among the pauper classes. Little is known positively on this subject. We know tolerably well the efficient causes, physical and moral, productive of lunacy in different classes of the population, but of the modes in which those causes are maintained in activity among the impoverished classes our knowledge is comparatively slight. The nature of the cases chiefly present in our public asylums would, apart from any other consideration, point to the propriety of an inquiry such as we speak of. These institutions, in consequence of the great and increasing accumulation of chronic cases of lunacy in them, answer but very imperfectly the principal object for which they were originally designed. Indeed, several of the largest asylums have become, from this cause, little better than houses of detention for the chronic and incurably insane. Sir Alexander Spearman, one of the visitors of the Hanwell Asylum, in his evidence before the Select Committee on Lunatics, said, respecting that asylum—

"There are numerous epileptic and paralytic patients, run-away patients, elderly and infirm patients, refractory patients, others in the infirmary, and quiet patients spread throughout the wards. . . .

Of acute cases there are few; of those requiring active medical treatment not many. . . . There are few curable patients. I asked Dr. Begley, yesterday, 'How many patients have you now under your charge who, you think, are in a state to be cured?' His answer was, 'I make a distinction always between the "possible" and the "probable." I think there may be 30 out of the 400 who may "possibly" be cured; but I do not think that there are more than 15 or 18 who will "probably" be cured.' *Mr. Tite.* Those are males?—Yes. The female patients are very much of the same character as those I have described; the epileptic and the paralytic, the refractory and some few run-away; many elderly and infirm, and some in the infirmary. I asked Dr. Sankey, the medical officer on the female side, the same question, and he told me he did not think that there were 10. I then said, 'If I am asked the question by the Committee to-morrow shall I be within bounds if I say that probably there are not more than 20?' He said, 'I think that there will not be more than 10, and you will be perfectly safe in saying that there are not more than 20 [that is, in about 600 patients].'"—(Queries 2858, 2859, pp. 237, 238.)

Mr. Cottrell, the Chairman of the Committee of Visitors to the Colney Hatch Asylum, said, respecting the patients in that asylum, in his evidence before the Select Committee:—

"—— (. . . As at Hanwell the proportion at Colney Hatch supposed to be susceptible of cure is very much the same) it must be remembered that we are an asylum rather than a hospital. We are a hospital only in occasional instances; an asylum always."—(Query 2946, p. 245.)

This state of things which is more or less common to all public asylums, is mainly due to the fact that the cases sent to them either are chronic when at first received, or they have at least passed beyond the stage which is most amenable to treatment. Two causes of delay in sending patients to asylums are patent: (1) a prior detention of the patient for a longer or shorter period in a workhouse; (2) the active dislike of sending friends or relatives to a *madhouse*, which is still prevalent among a large section of the population—a relic, in no small degree, of the just fear once entertained regarding institutions for the insane.

By the operation of these causes, the flood of chronic cases which inundated our public asylums when first opened, is kept up, and they are rapidly becoming more and more inoperative as curative establishments; and thus it happens that our best conceived plans hitherto have not touched the core of the evil to remedy which they were devised, and that at the best they are merely palliative.

Thanks to a recent inquiry, by the Commissioners in Lunacy, into the condition of lunatics in workhouses, the results of which inquiry have been made known in a Supplement to the Commissioners' Twelfth Report, we are enabled to judge with tolerable accuracy of the influence exerted by the detention of lunatics in these institutions, not only in clogging our asylums with chronic and incurable cases, but also in fostering pauper lunacy; and no better illustration could be given than the facts elicited by this inquiry of the practical importance of a full examination into the causes chiefly concerned in the maintenance of lunacy among the poor.

Let it be remembered, however, that pauperism is only one, and that the most immediately perceived, result of the prevalence of insanity among the impoverished classes of a population. Another, and more remote, but not less important consequence of insanity among these classes (as in all classes), is the influence which it exercises as a source of physical and moral deterioration. The transmission of special forms of insanity in their entirety from parents to children may be the chief, but it is not the sole fact of moment in the heritage of unsound mind; for in the offspring of the insane there is frequently recognised a deviation from healthy mental action, which, while it would not be denominated insanity, differs from it, perhaps, only in degree, and is not less important from its bearing upon the moral integrity of the subject. There may be a slight degree of imbecility, unusual susceptibility to emotions, impaired volition, or a perverted condition of the moral faculties. Just, indeed, as a child may inherit one or more lineaments of a parent, and these be more or less distinctly characteristic, so he may also inherit one or more phases of mental unsoundness, and these be manifested in a greater or less degree. Nor is this all; for it is found that the slighter derived deviations from a sound mental condition are apt in succeeding generations to be transformed into unmitigated insanity—exactly as in the hereditary transmission of physical defects it not uncommonly happens that they are but slightly manifested or not at all in one generation, while they reappear in full in a succeeding one. Of the physical degeneration connected with idiocy and imbecility we need say nothing.

When we find a combination of circumstances existing which give rise to a constant reproduction, by hereditary transmission, of unsoundness of mind, we may rest assured that we have there an important source of the physical and moral degradation of a people. We can mark clearly the beginning of the evil, but we cannot point out where it terminates.

Certain facts recorded by the Commissioners in Lunacy, in the Supplementary Report referred to, show that circumstances do exist which lead to the perpetuation of lunacy among the poorer classes of our population by hereditary transmission; which thus give rise to pauperism on the one hand, and to deterioration of the population both physically and morally on the other; and which point most clearly to the necessity for further inquiry into the fostering causes of lunacy among the poor.

We are told that cases like the following, in connexion with our workhouses, are not of unfrequent occurrence:—

"In the Newark Workhouse, among other instances, two females were met with during the past year, who, although classed as of weak mind, were in the habit of discharging themselves, and after a short absence returning in the family way. Each of them had three illegitimate children in the house; and it became the duty of the Visiting Commissioner strongly to urge upon the guardians the necessity of exercising the powers vested in them of absolutely refusing to allow these women again to quit the house. This, however, the guardians did not feel themselves justified in doing; and at a subsequent visit, in November last, one of these females had again been permitted to leave. In the Walsall workhouse we found an idiotic female who had had four illegitimate children; and in the Monmouth workhouse, two imbecile paupers, each of whom had had three illegitimate children, and one of whom was again pregnant. In the Tamworth workhouse there were two idiotic females, of whom each had a child.

"In addition to these cases, moreover, it is right to state that a far more painful instance of the evil of allowing inmates of weak mind to leave the house, and go out into the world unprotected, lately came under our notice in the Martley workhouse in Worcestershire. A female who had for some time been classed as of weak mind, was struck off the list in 1856, and was allowed to leave the house for the purpose of saving expense to the parish by earning something by hop-picking. This woman had previously had two illegitimate children by paupers in the house, one of whom had died; the other (a girl about 10 years of age), she took with her, on quitting the house, to her mother's home. When there, she and her daughter slept in the same room with her father-in-law and her mother, and in the same bed with two of her brothers. The result of this indecency was, that she returned to the workhouse in the family way, and was delivered of a child, the father of which she distinctly stated to be one of her brothers, but which of them she was unable to specify. This woman, though able to perform some useful work, was decidedly of weak mind; and there can be no doubt that, under the circumstances, the guardians were justified in detaining her in the workhouse, and that they ought not to have sanctioned her quitting it. Assuredly it would have been right to place her in the class of persons of weak mind on her return to the workhouse; but this was not done; and she only accidentally came under the notice of a member of this Board, to whom the foregoing particulars of her case were communicated. When it is remembered that the offspring of these weakminded females but too frequently inherit and communicate to their own children the imbecility of their mothers, the importance of more stringent regulations will at once be apparent. At a visit to the Calne workhouse, a few years ago, a member of this Commission saw three paupers, the grandmother, the mother, and the daughter, all imbecile, one or two, indeed, verging on idiotcy; and we have been assured that the same transmitted defect of intellect has been observed in the fourth generation."—(*Supplement to Twelfth Report*, pp. 15, 16.)

If the occasional inmates of workhouses furnish examples of this character, what may we expect to find among out-door pauper and indigent imbeciles? The question may be answered, in part, in regard to Scotland, for the Scotch Commissioners in Lunacy have instituted a special inquiry into the condition of out-door pauper and of indigent imbeciles and lunatics in that country; and they report of one county alone, that, of 349 single patients visited—

"One hundred and thirteen were females above seventeen years of age. Of these, 22 were in circumstances affording adequate protection to their chastity. Of the remaining 91, 15 were known to have given birth to illegitimate children, and 5 to have borne more than one child. Of the 15 mothers, 3 are known to have been illegitimate, and 12 are at present paupers; of their children, 6 are known to be idiots. There are, besides, in the county, 3 other idiots who are known to be the offspring of insane or imbecile mothers, who are dead or have disappeared."—(*First Report of the General Board of Commissioners in Lunacy for Scotland*, p. xi.)

In the *Appendix* to the same Report, the following, among other examples, are recorded:—

"(1.) M. H., a female; cannot tell her age. Congenital imbecility, a beggar, cottage, bedding, &c. dirty and squalid. Lives with two daughters and one granddaughter, all of whom are illegitimate, and, with the exception of the last, of weak mind. 1*s*. a week, rent paid, and a cart of coals.

"(2.) L. H., a female, daughter of the above, aged 30. Congenital idiot; ineducable. A beggar; condition wretched; lives with her mother in a miserable room. Has an illegitimate child, aged 5. No allowance from parish. . . . . .

"R. R., a male, aged 12. Imbecility; but of what degree could not be accurately ascertained, as the boy was not at home when sought, and his grandfather could not give the necessary information. Cottage small, squalid, and miserable, with two beds for six occupants. R. R. has two cousins in a state of imbecility. His mother was idiotic, wandered about the country, and bore three illegitimate children. R. R. receives 1*s*. 6*d*. per week.

"(1.) A. F., a female, aged 51. Imbecile; speaks; walks; deformed; affectionate, though at times she is cross and needs humouring; industrious; knits. In average bodily health. Tolerably well clothed. Had an illegitimate child, who is alive, and is said to help her a little. Sleeps alone, and has a good bed.

"(2.) R. F., a male, aged 38. Said to be idiotic; to speak imperfectly; to be much weaker in the mind than his sister (the case preceding); to be honest and sober; to be able to go messages; to be noisy and troublesome at times; not to sleep well to have a repulsive look, causing terror to those who do not know him; to be unsettled; to gain little; to be strong and able-bodied; to be cleanly in habits; to need help in dressing. Sleeps alone in a bed close to that of his imbecile sister, and no one else being in the room with them. Both live with their mother, a widow. There are seven inmates and four beds. Allowance, 39*s.* quarterly for the two.

"(3.) M. F., a female, aged 16. Idiot, squints, slavers; oscillates; does not speak; does not walk; needs help in dressing; harmless; not in robust health. Tolerably well clothed. Lives with grand-parents. Said to have been practically deserted by her father, who was reported to me as a drunkard. I do not believe that her grand-parents, who are paupers, have anything to live on but the parochial allowance, so that she, too, is virtually a pauper; but, as she is not reported as such, the Board have no jurisdiction in the case.

"This girl is a niece of the two preceding lunatics. In this family we find an imbecile daughter, with an illegitimate child; an idiot son; a married son, who is a drunkard, and has an idiot daughter; a sane daughter, with an illegitimate child; and a sane son with the same—illustrating the close connexion between intellectual and moral insanity."—(p. 195-196.)

That an inquiry into the condition of indigent and out-door pauper imbeciles and lunatics in England would make known a condition of things in reference to illegitimacy similar to that ascertained to exist in Scotland, the examples already quoted from the Supplementary Report of the English Commissioners in Lunacy renders only too probable; and in regard to Scotland it may be safely averred, that the insufficient protection of indigent and pauper female imbeciles and lunatics, and the illegitimacy arising therefrom, constitute a most formidable source of evil, active alike in the perpetuation of pauper lunacy, and in deteriorating both physically and morally a large section of the population. To what extent like circumstances may bring about similar results in England has still to be ascertained, and we insist upon the necessity of inquiry to determine this; for if we have here a powerful source of lunacy and its train of evils, we have likewise at our command, in so far as paupers are concerned, the means by which this source may be stopped almost altogether.

"Heretofore, in Scotland," saith Hect. Boëthius [who wrote three hundred years ago, be it borne in mind], "*if any were visited with the falling sickness, madness, gout, leprosie, or any such dangerous disease, which was likely to be propagated from the father to the son, he was instantly gelded; a woman kept from all company of men; and if by chance, having some such disease, she were found to be with child, she, with her brood, were buried alive;* and this was done for the common good, lest the whole nation should be injured or corrupted. A severe doom, you will say, and not to be used amongst Christians, yet more to be looked into than it is. For now, by our too much facility in this kind, in giving way for all to marry that will, too much liberty and indulgence in tolerating all sorts, there is a vast confusion of hereditary diseases, no family secure, no man almost free from some grievous infirmity or other. When no choice is had, but still the eldest must marry, as so many stallions of the race; or, if rich, be they fools or dizzards, lame or maimed, unable, intemperate, dissolute, exhaust through riot, (as he said) *jure hæreditario sapere jubentur;* they must be wise and able by inheritance; it comes to pass that our generation is corrupt; we have many weak persons, both in body and mind; many feral diseases raging amongst us, crazed families, *parentes peremptores;* our fathers bad; and we are like to be worse."—(*Burton's Anatomy of Melancholy, Pt. I., Sec. II., Mem.* 1. *Subs.* 6.)

We, fortunately, lost long ago the barbarism which characterized the remedies devised by the ancient Scots, but at the same time we well nigh lost all active regard of the principle which prompted the remedies attributed to them. They acted according to the light which they had; we have not acted according to the light which we have. Both humanity and policy point to the necessity for adopting such precautions, in regard to pauper lunatics and imbeciles, that they should neither beget, nor have begotten upon them, children; and we have the means justly at our disposal, by the exercise of a proper restraint and care, to ensure this. The Commissioners in Lunacy remark:—

"The absolute necessity of extending greater protection to idiotic and weak-minded women has uniformly been recommended by us. But although our efforts to inculcate the importance of due care in this respect has produced a certain amount of benefit, we find, from repeated examples, that the subject has not received the full consideration it deserves."—(*Supplementary Report, p.* 15.)

The condition of the insane in workhouses is a subject almost as grave as that we have just noticed; for from the Report of the Commissioners in Lunacy it would appear that our workhouses, in so far as lunatics are concerned, are little better than *hot-beds* in which pauper lunacy is fostered and matured. This is a serious charge, but it is supported by weighty evidence. In the course of 1857-1858, the Commissioners visited and inspected the whole of the workhouses (655 in number) in England and Wales, and this visitation has disclosed that, in the majority of instances, the lunatics detained in them are confined in unsuitable apartments; that they are deprived of all exercise; that they are badly fed and badly clothed; that they have no proper attendance either medical or general; that they are subjected to harsh mechanical restraint when violent; and that, as a consequence of this entire absence of necessary care and treatment, recent cases of lunacy

(which are frequently confined in workhouses in defiance of the law) are matured and confirmed, this being, as the Commissioners state, "*one of the most fertile causes of the increase of lunatic paupers throughout the country. It is this that mainly tends to fill our county asylums with hopeless chronic cases, and is most directly responsible for the heavy and permanent burdens upon the parish rates.*"—(p. 34.)

The cases chiefly met with in workhouses consist of persons suffering from chronic dementia, and from melancholia, of epileptics, idiots, and imbeciles. But during late years cases of acute insanity have been frequently met with, especially in the larger houses.

Both the management and the internal economy of workhouses are, however, inconsistent with a due care of almost every grade of lunatics; and even when the lunatics are confined in special wards, the evils are rather augmented than diminished. A reduced diet, task labour, and confinement within the narrow limits of the workhouse are thought to be necessary, as well to test the actual wants of those applying for admission as to check abuse and imposition as far as possible; but a scanty diet and restricted exercise not only tend to increase the mortality of the insane, but to prolong and confirm the disease. The rules in force for the government of workhouses are devised to check disorderly conduct among ordinary paupers, but they are too commonly applied to the imbecile and those of unsound mind:—

"Any increase of excitement, or outbreak of violence, occurring in the cases of such patients, instead of being regarded as a manifestation of diseased action requiring medical or soothing treatment, has subjected the individual to punishment, and in several instances led to his imprisonment in a gaol."—(p. 7.)

Of separate lunatic wards connected with workhouses, the Commissioners say:—

"Some have existed for a considerable time, and others are of recent construction. In some of the wards attached to the old workhouses, the rooms are crowded, the ventilation imperfect, the yards small and surrounded by high walls; and in the majority of instances the bedrooms are used also as day-rooms. In these rooms the patients are indiscriminately mixed together; and there is no opportunity for classification. There is no separation where the association is injurious; and no association where such would be beneficial. In fact, patients of all varieties of character, the weak, the infirm, the quiet, the agitated, the violent and vociferous, the dirty and epileptic, are all mingled together, and the excitement or noise of one or more injures and disturbs the others. The restless are often confined to bed to prevent annoyance to the other patients, and the infirm are thus disposed of for the want of suitable seats. Their condition when visited in the daytime is obviously bad, and at night must be infinitely worse.

Even in workhouses where the wards are so constructed as to provide day-rooms, these are often gloomy, much too small in size, and destitute of ordinary comforts; while the furniture is so poor and insufficient, that in some instances, there being no tables whatever, the patients are compelled to take their meals upon their knees. Other cases to be hereafter mentioned will indeed show that it is reserved for lunatic wards of this description, and now happily for them only, to continue to exhibit some portion of that disregard of humanity and decency which at one time was a prevailing characteristic in the treatment of insanity.

"Occasionally, indeed, in those workhouses where lunatic wards are of recent construction, the accommodation for patients is better; but they all want the continued superintendence of a resident medical officer and the assistance of a sufficient staff of properly qualified attendants, and they are greatly deficient in reference to diet, exercise, occupation, and general arrangements. For the most part, the rooms are gloomy and prison-like, and considerable expense has been repeatedly incurred in formidable contrivances to prevent escape or accident, which any proper system of nursing and attendance would have rendered quite unnecessary."

There is no proper supervision medical or otherwise, neither is there any record kept of treatment or restraint; and there is no efficient or authoritative visitation of the lunatic wards by the visiting justices, and the visits of the Commissioners in Lunacy are almost useless, except as enabling them to detect the evil that exists at the time of the visit, and which, after all, they have no power to remove.

The detention of a lunatic in a workhouse rests entirely upon the caprice of the parochial authorities, for he is confined without either medical certificate or magistrate's order, and consequently without that protection which is extended to the lunatic in public and private asylums. Certainly his detention in a workhouse at all is in a great measure illegal, but the law is inoperative, from various causes, as we shall see presently.

In sending a lunatic from a workhouse to an asylum, unmanageableness would appear to be the principal criterion adopted by the parochial authorities as to its advisability. The Commissioners state that:—

"An impression frequently prevails that if a patient be quiet there is no necessity for his removal to an asylum; and, urged by considerations of economy, guardians constantly act upon this impression. It is an error pregnant with the most serious evils. Those who suffer most are often the least complaining. In a very recent case of semi-starvation at the Bath Union, when the frauds and thefts of some of the attendants had, for a considerable time, systematically deprived the patients of a full half of their ordinary allowance of food, the only complaint made was by the wan and wasted looks of the inmates. The melancholic and taciturn especially, when (as is often the case)

their physical condition is enfeebled by long privation, remain quietly suffering until their malady becomes confirmed and incurable. Placed in gloomy and comfortless rooms, deprived of free exercise in the open air, and wanting substantial nutriment sufficient to promote restoration, they pass their lives in a moody, listless, unhealthy, inactive state, which is fatal to their chance of ultimate recovery.

"For cases like these a workhouse is the most unfit, and the asylum the most proper place; and the error of considering manifestations of violence, excitement, or dangerous propensities, as the only or principal reasons for removing a patient from a workhouse to an asylum, cannot be too widely denounced.

"The chronic and less hopeful patients, in like manner, who have become insensible to their ordinary wants, and inattentive to the calls of nature, are most unfortunately situated when detained in a workhouse. Little or nothing being done to revive a sense of decency by vigilant attention and judicious care, they sink into deplorable discomfort, and exhibit the lowest state of mental and bodily degradation."

To illustrate some of the foregoing points more particularly—

The importance of a good nutritious diet in the treatment of the insane need not be insisted on. The Commissioners speak of the evil consequences which have resulted from the scanty diet of workhouses when it has been necessary, on account of overcrowding, to remove chronic cases to workhouses from asylums, a marked deterioration in their condition following, and at times harmless patients have been rendered so irritable and violent as to render it necessary to replace them in an asylum. As examples of the character and variations of workhouse dietaries, the Commissioners state that:—

"While the diet of the insane in the Brighton workhouse is very substantial and liberal, the diet given to the same class in the Hailsham Workhouse (in the same county) contains but one spare meat dinner during each week, the proportion of meat allowed being only 4 oz. for men and $2\frac{1}{2}$ oz. for women; while bread and cheese, without beer, constitutes the dinners on four other days, and pudding on the remaining two. At the Freebridge Lynn workhouse, Norfolk, meat is allowed only once a week, bacon once, and bread and cheese constitutes the dinner for the remaining five days: yet, in the King's Lynn workhouse, in the same county, meat is allowed for dinner during three days, suet pudding twice, and good soup or rice milk twice. In many other workhouses, meat is only given to the insane once a week, and even then in a very small and insufficient quantity. In the West Firle workhouse, within a few miles of Brighton, the allowance is only three ounces of meat during the whole week for each inmate. In the Amesbury workhouse, the inmates are restricted to bacon twice a week (four ounces for men, and three ounces for women), pudding twice, and a very weak soup twice. It is at the same time right to add that in some workhouses the diet is on a more liberal scale, meat being allowed three or four days a week, and soup of fair quality on other days. In a very small number of houses a meat dinner is given every day."

As to medical treatment we are told, as an example of its total inadequacy, that at the Leicester Union the medical officer visits the lunatic wards once a quarter.

The attendants are paupers, not unfrequently old and feeble people, to whom are entrusted means of restraint, and who in return for their labour receive occasionally some trifling gratuity, such as an allowance of beer or spirituous liquors, or an increase of diet.

"1. In the Stepney Union, at Wapping, the nurse is a pauper receiving no pay, but having extra food, and two pints of beer daily. The matron said that she was kind towards the patients (30 in number); but her manner certainly did not indicate kindness. Inquiries being made as to the diet, she could not (although she asserted she knew 'all about it') tell the amount of food at any meal. After repeated mistakes in giving the names of the patients, she insisted that she went 'entirely by her list.' On the list being produced, it was found that she could not read (November, 1857).

"2. In the St. Alban's workhouse, the nurse having the care of the insane females was herself decidedly of unsound mind. She was allowed half a pint of beer daily, and no other remuneration for her services. As she was of weak intellect and very excitable, the Visiting Commissioner strongly urged the necessity of the guardians engaging a competent paid attendant (30th December, 1857). This suggestion, however, it was found at the last visit, had not been attended to.

"3. In the St. Martin's workhouse, a violent lunatic was entrusted to the supervision of an old pauper, 70 years of age ('the keeper of Ward F.') The patient appears to have been placed in a room, in which were a poker and other dangerous articles; and on being relieved from some restraint which had been imposed upon him, he struck the feeble old pauper on his head until he died (July, 1857).

"Without giving further instances, we may confidently state, that, as a general rule, the attendance and nursing in workhouses are totally inadequate."

Concerning the accommodation for lunatics the Commissioners state that—

"In many instances the crowding is excessive; the ventilation of the rooms imperfect; and the furniture, even of the poorest description, scanty and insufficient. The deficiency of tables, and of seats for the helpless and feeble, is almost constantly observed; and the want of such suitable seats frequently renders it necessary to keep patients always in bed. Even articles of furniture indispensable to order, decency, and cleanly habits, are often most sparingly supplied, or altogether wanting. In the Blackburn workhouse, we found the small day-rooms on the male side incumbered with large iron guards and heavy restraint chairs; and in one room on the women's side, used both as a bed and day-room, the beds were until recently so close as almost to touch each other, and a large portion of one apartment containing beds was boarded off as a privy. In addition to similar discreditable arrangements, the patients have frequently no means of bathing, and very scanty means are provided for washing their persons. A tub frequently supplies the place of chamber utensils in the dormitories; and we have ascertained that in some cases the vessels which are used as urinals in the night time serve for the patients to wash, or be washed in, in the morning. It rarely happens, indeed, that patients in workhouses have any proper means of washing themselves near their bedrooms; a trough or sink, common to all, being for the most part substituted for basins. Occasionally, the patients wash out of doors at the pump, or in tubs or bowls placed, whatever the season, in an outhouse, or an open shed."

The clothing is wanting in warmth, and the bedding is too often dirty and insufficient, while in some workhouses the most objectionable practices exist with regard to the night arrangements for the lunatics. At the Huddersfield workhouse, for instance—

"The evils arising from the detention of insane patients in a workhouse have been made manifest to a frightful extent, owing to the shamefully defective accommodation provided in that Union. It will be seen from the subjoined reports that no less than one-third of the inmates are insane or idiotic; that many of them are dirty in their habits, and placed to sleep together sometimes even in a perfect state of nudity. The bedding is stated to be both scanty and filthy, and the floors of the dormitories to be saturated with urine.

"There being no bath-room, lavatory, washhouse, or laundry on the premises, a small room on the ground floor is used for these purposes, and also as a kitchen, a brewhouse, and a bakehouse. Over the room serving these seven distinct objects, are the dormitories appropriated to the sick; the atmosphere of which is both over-heated and contaminated by noxious emanations escaping from below; and also from the wet clothing occasionally hung up to dry around the bed-ridden patients.

"Those idiotic or insane inmates not bodily disabled are crowded together and confined in a very limited space, they are associated with most abandoned characters, secluded in the *dead-house*, and are so circumstanced in other respects, both during day and night, as to aggravate their mental infirmities, and lead to a deplorable state of degradation."*

Restraint chairs, chains, handcuffs, leg locks, muffs, straps, and strait-waistcoats are in frequent use, and are applied by the attendants without the sanction, and probably without the knowledge of the medical officer or master of the workhouse. We read of lunatics chained to benches, and strapped to their beds at night

* Mr. Farnall, the Inspector of the Poor in the Metropolitan district, in his evidence before the recent Parliamentary Committee on Lunatics, stated that he had heard that the Huddersfield guardians had "come to a vote to build a new workhouse, *but not a sufficient one; we have no power to order them to do it.*"—(*See Report.* Query 1609.)

in the Dewsbury workhouse, "and the only reason assigned for this treatment was, that some would run away, and others get out of bed." In the Bury workhouse the Visiting Commissioner found two lunatics strapped to the bedsteads, and one with his hands secured in a leather muff, while iron staples, shackles, chains, and cord were found in one of the men's wards, "all evidently intended to be employed for the purpose of fastening down violent patients." At the Llanelly workhouse a female lunatic was found most ingeniously, but most cruelly, fastened to a bedstead, upon which it was customary to restrain her for periods varying from a few days to a week.

"The patient was undoubtedly a dangerous lunatic, and the Visiting Commissioner recommended that she should be at once removed to an asylum. The guardians, however, persisted in an attempt to justify their proceedings, and to prove her harmless; and they obstinately refused to remove her according to recommendation."—(p. 26.)

Three females were found in bed, restrained by strait-jackets, in the workhouse of St. George the Martyr, Southwark. These unfortunates were thus restrained day and night, and had been so for some time previously.

Several examples are given of the insufficiency of the space connected with workhouses for the proper exercise of patients. Thus—

"In the Clerkenwell workhouse the lunatic wards are nearly at the top of the house, rendering it impracticable for infirm patients to gain the small yard below, which is long and narrow, and bounded on each side of its length by high buildings. This small yard is nevertheless the only place allotted for exercise to the 511 paupers in the house (November, 1857). . . . . In the Bolton workhouse the greater portion of the paupers (343 in number) are crowded together in such a way as to deprive them of all comfort. The yard for the two lunatic wards is quite useless for the purpose of exercise, being scarcely larger than a good-sized room (March, 1855)."—(p. 28.)

We need not comment on the irritating and injurious influence, or on the misery inflicted upon lunatics, by confining them within doors or within a limited area; but an example of one of the parochial methods of dealing with irritated and irritable lunatics deserves quotation:—

"At a visit to the Downham workhouse, the case of a male prisoner then in Swaffham Gaol was brought under notice. He had been recently an inmate of the workhouse, and was classed as of unsound mind, yet had been permitted repeatedly to discharge himself. Upon his last quitting the workhouse, he was soon afterwards sent to gaol for six months for an indecent assault upon a female. He had previously been three times in the County Lunatic Asylum, and was there described as dangerous to others. Whilst in the workhouse he was thought scarcely responsible for his actions, yet had been committed to gaol on fourteen occasions for refractory conduct. The Governor thought he was not a proper subject for punishment in prison, in which opinion the Visiting Commissioner concurred. The attention of the Visiting Justices being drawn to the case, they agreed that it was not right to subject the man to punishment; but as the surgeon declined to certify his insanity, they suggested that the matter should be referred to the Secretary of State, which was done. On the ground, however, of the medical officer of the gaol still declining to certify, the Secretary of State did not feel justified in interfering in the case."

The congregation and detention of lunatics in workhouses is, doubtless, due in part to the insufficiency of asylum accommodation; but the chief evil arising out of the detention, the impediment cast in the way of an early and proper treatment of many cases of insanity in asylums, arises mainly from an evasion of the law.

In providing for the erection of county and borough asylums, the law directed that all pauper lunatics should be placed there at the outset of their malady; and it imposed certain duties, requisite for the effective carrying out of this wise provision, upon parochial authorities. These duties are, however, continually neglected or evaded, particularly in the more populous districts; and the principal object of the asylum as a curative establishment, and the welfare of the patient are, in consequence, sacrificed.

"The law directs that every pauper deemed to be lunatic, and proper to be sent to an asylum, shall be taken before a justice, who, upon being satisfied that he is a fit person to be taken charge of and detained, shall order his removal to the County Asylum. But instead of this course being adopted, it is now almost universally the custom to remove the patient in the first instance to the Union Workhouse; where, if he appears to be quiet and harmless, he is suffered to remain.

"Instead of causing the patient to be dealt with as directed by the 67th and 68th sections of the Lunatic Asylums Act, 1853, and immediate steps to be taken for his direct removal to the asylum, workhouses have been to a great extent made use of primarily as places for the reception, and (in many instances) for the detention, of recent cases of insanity.

"The workhouse is thus illegally made to supply the place of a lunatic establishment, and the asylum, with its attendant comforts and means of cure, which the law has provided for the insane poor, is altogether disregarded; or it comes into operation only when the patient, by long neglect, has become almost hopelessly incurable."

The phraseology of the Acts relative to the detention of lunatics in workhouses admits, however, of ready evasion of their provisions, and do not, indeed, apply to a large and most important class of lunatics; and until this defectiveness of the Acts be removed, we see little hope of escape from many of the troubles connected with the removal of patients to asylums. The 45th section of the Act 4 and 5 Will. IV. c. 76, provides that "every person wilfully detaining in any workhouse any dangerous lunatic, insane person, or idiot, for more than fourteen days, shall be deemed guilty of misdemeanour;" and Article 101 of the General Consolidation Order of the Poor-Law Board directs "that no pauper of unsound mind who may be dangerous, or who may have been reported as such by the medical officer, or who may require habitual or frequent restraint," shall be retained in a workhouse for more than fourteen days. These provisions, it is clear, are not applicable in the slightest degree to unsoundness of the mind unaccompanied by violence.

We have already pointed out the influence exercised by the detention of lunatics in workhouses in fostering pauper lunacy, and in filling our asylums with chronic cases; and, on the other hand, it may be understood how the diminution of the curative power of the asylums from this cause reacts upon the workhouses, and tends to keep up and increase the accumulation of lunatics there. This reaction of the one class of institutions upon the other; the consequent impairment of the most beneficial effects of the asylums; and the augmentation both in workhouses and asylums of matured and confirmed lunatics, might be supposd to be a sufficient argument against the folly of those parochial authorities, who conceive that they practise a true economy by detaining their insane in workhouses. But, in addition, the Commissioners express the opinion that the difference of cost between the maintenance of a lunatic in an asylum and in a workhouse is not so great as is ordinarily conceived, for—

"In the mode of apportioning the cost of a pauper in a workhouse, several items are excluded from the maintenance account which, in asylums, appear to render that account considerable. In the former case, food and clothing are generally the main items of outlay; whilst in the other all salaries, and many articles entitled 'necessaries,' are included. These latter expenses in parishes are passed over to the Union fund, and paid from a different source. The ostensible cost, therefore, of the patient's 'maintenance' in a workhouse does not represent the same expenses as his maintenance account in an asylum.

"In addition to this, it is also to be observed that the inmates of workhouses consist, in a great measure, of children and aged persons, who are maintained at a small cost. Therefore, when an average of the entire outlay is struck, and an equal charge made for every occupant, whether expensive or otherwise, the actual cost of the insane patient (which far exceeds that of the rest) does not appear."

To remove the evils connected with the detention of lunatics in workhouses the Commissioners offer certain suggestions. The first of these is based upon an opinion of the entire unfitness of workhouses, from their character and management, for the proper care of lunatics—an opinion concerning which little doubt can be entertained. They suggest that inexpensive buildings should be erected in connexion with, or at a convenient distance from existing asylums, for the reception of chronic, idiotic, and harmless cases. These auxiliary asylums need not cost half the expense in building and management of our ordinary asylums, and they would serve the purpose of relieving workhouses entirely, and asylums mainly, of their chronic cases, and leave the latter free to develope fully their capacities as hospitals.

Some such plan must ultimately become necessary, as it is very evident that our present system of asylum accommodation cannot continue much longer without coming to a dead-lock. But we insist upon the importance of ascertaining fully the amount and character of the evil we have to contend with before any plan of additional asylum accommodation is carried out on a large scale; because, from what we have already said, it is evident that it is not sufficient to base future modifications of our plans for the care and treatment of lunatics on the existing pauper lunacy in our workhouses and private asylums (for the Commissioners have pointed out the fitness of removing the pauper inmates of private asylums as well as the lunatics in workhouses). There can be little doubt that as full an inquiry into the condition of pauper and indigent lunatics, idiots, and imbeciles outside the walls of asylums and workhouses, as that which has taken place into the condition of lunatics within workhouses, would lead to as valuable results.

The further suggestions of the Commissioners are well calculated to improve the state of lunatics in workhouses, so far as that is susceptible of improvement, until such a period as a scheme may be devised and carried out which will close those buildings altogether to lunatics except for very temporary purposes. They say:—

"We think it essential that Visiting Justices of Asylums should be invested with full power, by themselves or their medical officers, to visit workhouses, and to order the removal of insane inmates therefrom to asylums at their discretion. They should also be empowered, upon the report of the Commissioners, to order the removal into the asylum of pauper patients boarded with strangers.

"No lunatic or alleged lunatic to be received into or detained in a workhouse, unless he shall have been duly taken before a justice or officiating clergyman, and adjudged by him as not proper to be sent to an asylum.

"In any case, however, wherein an order for a lunatic's reception into an asylum shall be made by a justice or officiating clergyman, it shall be competent to him, if, for special reasons to be set forth in his order, he shall deem it expedient, to direct that such lunatic be taken, *pro tempore*, to the workhouse, and there detained for such limited period, not exceeding two clear days, as may be necessary, pending arrangements for his removal to the asylum.

"A list of all inmates of unsound mind to be kept by the medical officer of a workhouse, and left accessible to the Visiting Commissioner.

"The medical officer to specify, in such list, the forms of mental disorder, and to indicate the patients whom he may deem curable, or otherwise likely to benefit by, or in other respects proper for, removal to an asylum.

"The Visiting Commissioner, and the Poor-Law Inspector, to be empowered to order and direct the relieving officer to take any insane inmate before a justice, under the provisions of the 67th Section of the Lunatic Asylums Act, 1853.

"In all cases of inmates of unsound mind temporarily detained in workhouses, the medical officer to be invested with full powers as respects classification, diet, employment, and medical and moral treatment, and otherwise."

In the preceding article we have endeavoured to show from the statistics of pauper lunacy, and the Report of the Commissioners in Lunacy on the condition, character, and treatment of lunatics in workhouses, the necessity which exists for a full inquiry into the amount and fostering causes of lunacy among the poor and indigent classes of the population, and while doing this we have examined the Report at a sufficient length we trust to aid in the immediate object at which it aims. The approaching Census, we have pointed out, would be the best, perhaps the only means, of ascertaining accurately the amount of lunacy among the poor and indigent classes, this inquiry constituting a portion of an inquiry into the entire amount of insanity in the kingdom—a desideratum in social as well as medical science; while a specific inquiry would be needed into the fostering causes of insanity among the poor. Whether this latter inquiry would be consistent with the onerous duties of the Commissioners in Lunacy, or would require for its carrying out a special Commission we cannot say; but we would again urge the importance and necessity of both the investigations we have indicated.

---

